# Supplementary figures and images for: A high-throughput behavioral screening platform for measuring chemotaxis by C. elegans
Source: PLoS Biol. 2024 Jun 27;22(6):e3002672. doi: 10.1371/journal.pbio.3002672 (PMC11210793; doi:10.1371/journal.pbio.3002672)

# S1 Figure

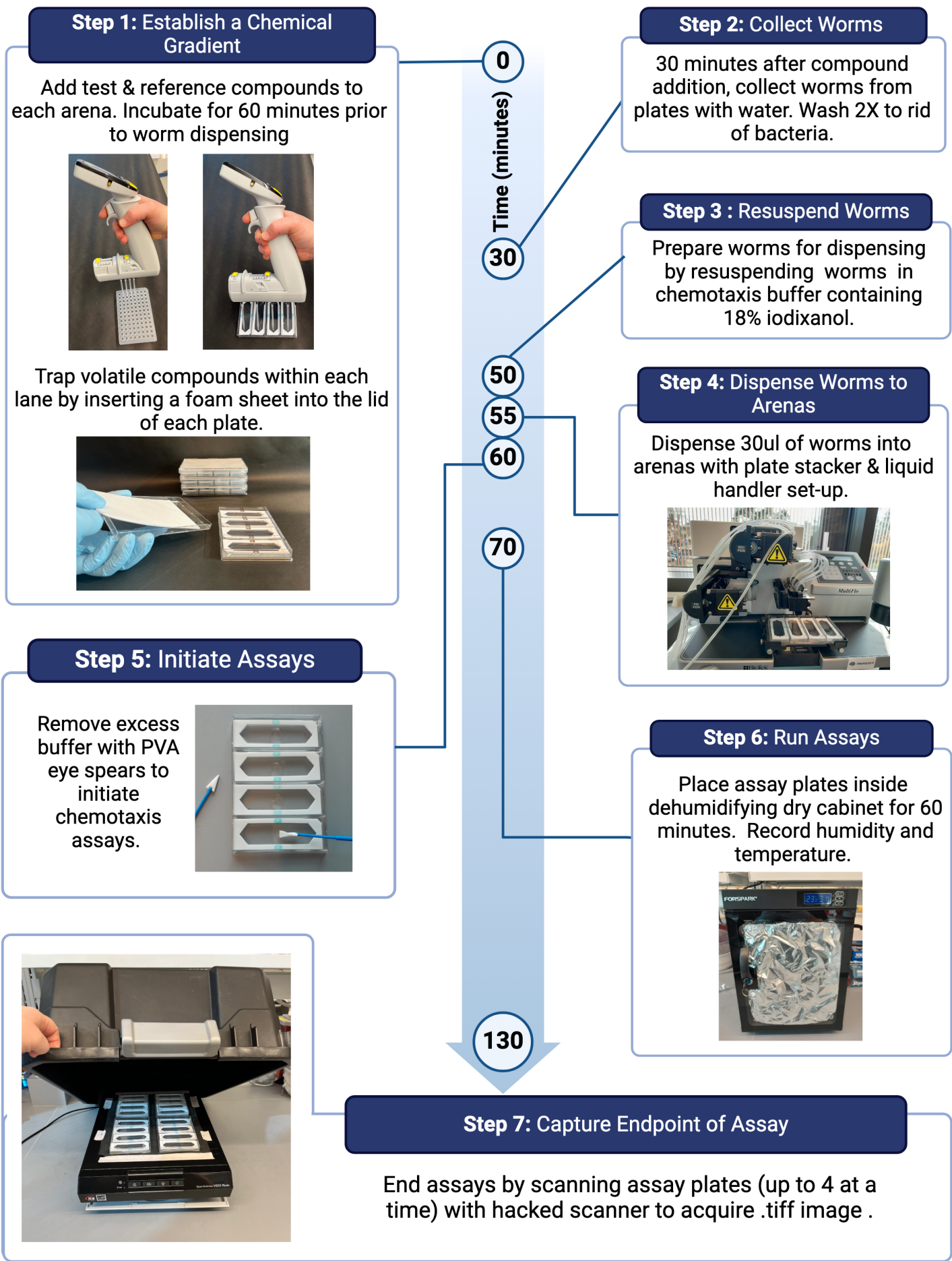

Supplement: S1 Fig — Timeline (in minutes) shown from top to bottom, time points (circles) and actions are indicated to the left and right of the timeline. Created with BioRender.com. (PDF) [file pbio.3002672.s001.pdf]

**S2 Figure**

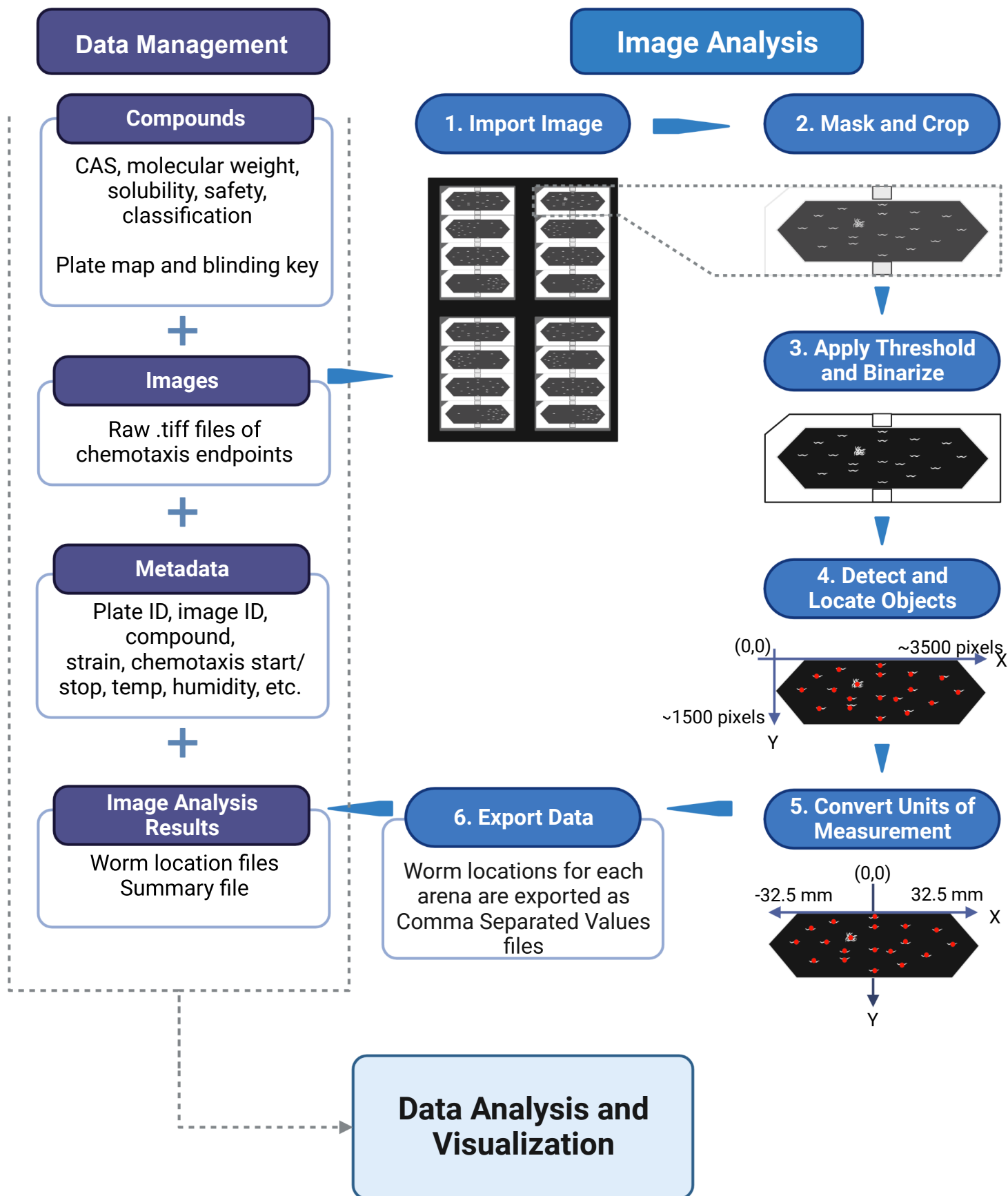

Supplement: S2 Fig — Data management (left) and image analysis (right) for the screens occur simultaneously, reducing data processing time, reducing data processing errors, and increasing reproducibility. Created with BioRender.com. (PDF) [file pbio.3002672.s002.pdf]
