# Supplementary material for: A high-throughput behavioral screening platform for measuring chemotaxis by C. elegans
Source: PLoS Biol. 2024 Jun 27;22(6):e3002672. doi: 10.1371/journal.pbio.3002672 (PMC11210793; doi:10.1371/journal.pbio.3002672)
Supplement: S1 Table — List of small molecules comprising the curated screening library, including the CAS registry number (aka CAS No.), common name used in this study, vendor, and catalog number. Vendors are (alphabetical order): Ambeed, Arlington Heights, IL; Aobious, Gloucester, MA; Cayman Chemical, Ann Arbor, MI; Chem-Impex, Woodale, IL; MCE = MedChemExpress, Monmouth Junction, NJ; Sigma-Aldrich, St. Louis, MO; TargetMol, Boston, MA; TCI = TCI America, Portland, OR; VWR International, Radnor, PA. Compounds generating visible precipitates in assay arenas are indicated with “(p)”. (PDF) [file pbio.3002672.s003.pdf]

S1 Table

CAS ID	Compound		Vendor	Catalogue #
0431-03-08	Diacetyl		TCI	B0682
0483-04-5	Ajmalicine		Cayman	31213
0491-09-8	Piperitenone		Cayman	25752
100-09-4	p-Anisic acid		MCE	HY-N1394
100-66-3	Anisole		Sigma	123226
102518-79-6	(-)-Huperzine A		MCE	HY-W019711
104-54-1	Cinnamyl Alcohol		MCE	HY-Y0078
104-87-0	p-Tolualdehyde		MCE	HY-W012860
105-87-3	Geranyl Acetate		Ambeed	A404343
106-22-9	Citronellol		MCE	HY-W010201
106-22-9	β-Citronellol		TCI	C0370
107-35-7	Taurine		MCE	HY-B0351
110-02-1	Thiophene		TCI	T0223
111-87-5	1-octanol		Sigma	297877
112-14-1	Octyle acetate		TCI	A0042
112-39-0	Methyl palmitate		TCI	P0006
116-26-7	Safranal		MCE	HY-N7560
1180-71-8	Limonin		MCE	HY-17411
119-65-3	Isoquinoline		MCE	HY-W012732
120-57-0	Piperonyl Alcohol		Sigma	P49104
123-11-5	p-anisaldehyde		TCI	A1674
123-35-3	Myrcene		Sigma	M100005
123-51-3	Isoamyl alcohol		Sigma	W205710
124-20-9	Spermidine		MCE	HY-B1776
126-17-0	Solasodine	(p)	MCE	HY-N0068
137-32-6	2-Methyl-1-butanol		Sigma	133051
14371-10-9	trans-Cinnamaldehyde		MCE	HY-W019711
1490-04-6	Menthol		MCE	HY-N1369
150-86-7	Phytol		MCE	HY-N3075
16409-43-1	L-Mimosine		Apex	B4751
168316-95-8	Spinosad	(p)	Cayman	25649
18524-94-2	Loganin		MCE	HY-N0512
18836-52-7	Pellitorine	(p)	Cayman	11662
19431-84-6	Curcumenol		MCE	HY-N2259
20283-92-5	Rosmarinic acid		MCE	HY-N0529
2068-78-2	Vincristine (sulfate)		MCE	HY-N0488
2244-16-8	(+)-Carvone		Sigma	22070
23180-57-6	Paeoniflorin		MCE	HY-N0293
23800-56-8	Pogostone		MCE	HY-N1416
24393-56-4	Ethyl p-methoxycinnamate		Cayman	11740
24697-74-3	Leonurine		MCE	HY-N0741
2482-00-0	Agmatine		Chemimpex	10668
3387-41-5	Sabinene		MCE	HY-108943
357-70-0	Galanthamine		MCE	HY-76299
3650-09-7	Carnosic acid		MCE	HY-N0644
372-75-8	L-Citrulline		MCE	HY-N0391
37839-63-7	Germacrene D		Aobious	CFN93281
4180-23-8	Trans-Anethole		MCE	HY-N0367

CAS ID	Compound		Vendor	Catalogue #
4373-41-5	Maslinic acid	(p)	MCE	HY-N0629
462-94-2	Cadaverine		Sigma	52063
464-45-9	(-)-Borneol		TCI	B1012
4674-50-4	Nootkatone		MCE	HY-N2195
469-61-4	(-)-Cedrene		MCE	HY-135190
469-83-0	Cafestol		MCE	HY-N6257
470-82-6	Eucalyptol		Sigma	C80601
474-58-8	Daucosterol	(p)	MCE	HY-N0410
474-58-8	Sitogluside	(p)	Targetmol	T3871
476-66-4	Ellagic acid	(p)	MCE	HY-B0183
484-20-8	Bergapten	(p)	MCE	HY-N0370
489-84-9	Guaiazulene		Cayman	31506
490-79-9	2,5-Dihydroxybenzoic acid		MCE	HY-W001179
496-16-2	Coumaran		MCE	HY-75247
496-16-2	2,3-Dihydrobenzofuran		TCI	D1583
499-75-2	Carvacrol		MCE	HY-N0711
508-02-01	Oleanolic Acid	(p)	MCE	HY-N0156
520-18-3	Kaempferol		MCE	HY-14590
520-36-5	Apigenin	(p)	MCE	HY-N1201
522-17-8	Deguelin	(p)	MCE	HY-13425
532-11-6	Anethole trithione	(p)	MCE	HY-B1223
536-74-3	Phenylacetylene		Sigma	117706
5451-09-2	5-Aminolevulinic acid (hydrochloride)		MCE	HY-W000450
55396-45-7	2-Nonylquinolin-4(1H)-one	(p)	Cayman	9003627
5784-74-7	Salsolidine		MCE	HY-22385
58-08-2	Caffeine		Sigma	C0750
5957-80-2	Carnosol		MCE	HY-N0643
6080-33-7	Sinomenine hydrochloride		MCE	HY-15122
628-97-7	Ethyl palmitate		MCE	HY-N2086
646-23-1	Alyssin		Cayman	31513
67-68-5	DMSO		VWR	N182
68370-47-8	Micheliolide		MCE	HY-N0847
689295-71-4	Salvinorin A Propionate	(p)	Cayman	22290
69-72-7	Salicylic acid		MCE	HY-B0167
70-26-8	L-Ornithine		MCE	HY-B1352
7212-44-4	Nerolidol		MCE	HY-N1944
76-22-2	Camphor		MCE	HY-N0808
77-52-1	Ursolic acid	(p)	MCE	HY-N0140
821-55-6	2-nonanone		TCI	N093
83-34-1	Skatole		MCE	HY-W007355
83-79-4	Rotenone	(p)	MCE	HY-B1756
84-79-7	Lapachol		MCE	HY-N6961
87-44-5	Beta caryophyllene		TCI	C0796
94-62-2	Piperine	(p)	MCE	HY-N0144
98-01-1	Furfural		TCI	F0073
98-86-2	Acetophenone		MCE	HY-Y0989
99-83-2	α-Phellandrene		Cayman	23179
	Water		NA	NA
