# Supplementary material for: A high-throughput behavioral screening platform for measuring chemotaxis by C. elegans
Source: PLoS Biol. 2024 Jun 27;22(6):e3002672. doi: 10.1371/journal.pbio.3002672 (PMC11210793; doi:10.1371/journal.pbio.3002672)
Supplement: S2 Table — Tabulated list of the difference of the mean position for wild type between each test condition and a reference condition (aka “mean difference”), sample size (n = worms pooled across N = 3 biological replicates), 95% confidence intervals for the mean difference (5% CI, 95% CI), and statistical testing (exact p-values, B-H correction for multiple comparisons with a false discovery rate of 5%). Control sample size was n = 1,065 for DMSO:DMSO, and n = 915 for DMSO:water for the respective comparisons. Mean differences and confidence intervals obtained by bootstrapping using the Dabest statistical package [43]. These data are shown graphically in Fig 5 and are from assays conducted with wild-type (N2, Bristol) adult worms. (PDF) [file pbio.3002672.s004.pdf]

Test_compound	Reference	Test_n	Mean_difference	CI_5	CI_95	p_value	BH_correction_0.05
Isoamyl alcohol	DMSO	502	14.167	12.82	15.512	1.35E-94	0.0005
2-Methyl-1-butanol	DMSO	381	11.712	9.891	13.409	6.39E-39	0.001
Thiophene	DMSO	706	9.76	8.202	11.263	7.61E-36	0.0016
*2,3-Dihydrobenzofuran	DMSO	705	9.194	7.63	10.674	2.43E-32	0.0021
Diacetyl	DMSO	796	8.019	6.59	9.361	7.99E-30	0.0026
Phenylacetylene	DMSO	496	6.45	4.656	8.165	5.75E-13	0.0042
Paeoniflorin	DMSO	556	5.382	3.587	7.05	1.12E-09	0.0073
alpha-Phellandrene	DMSO	777	5.368	3.801	6.836	4.13E-12	0.0047
Acetophenone	DMSO	755	5.238	3.649	6.776	5.14E-11	0.0052
*Coumaran	DMSO	754	4.93	3.386	6.361	8.19E-11	0.0057
Leonurine	DMSO	766	4.534	3.003	6.077	7.39E-09	0.0078
Guaiazulene	DMSO	958	4.305	2.961	5.685	5.86E-10	0.0063
L-Mimosine	DMSO	916	4.282	2.806	5.731	9.49E-09	0.0083
Solasodine (p)	DMSO	826	3.821	2.257	5.414	2.10E-06	0.0099
Isoquinoline	DMSO	836	3.807	2.368	5.358	5.97E-07	0.0094
Furfural	DMSO	796	3.619	2.048	5.138	4.40E-06	0.0104
(-)-Huperzine A	DMSO	911	3.378	1.87	4.772	5.08E-06	0.0109
Anisole	DMSO	858	3.017	1.461	4.546	1.27E-04	0.012
Limonin	DMSO	907	2.823	1.348	4.346	2.23E-04	0.0141
Cinnamyl alcohol	DMSO	857	2.814	1.287	4.297	2.47E-04	0.0146
Piperitenone	DMSO	951	2.707	1.319	4.16	1.88E-04	0.0135
Ethyl palmitate	DMSO	887	2.574	1.056	4.11	9.54E-04	0.0161
Carnosol	DMSO	930	2.521	1.026	3.964	7.70E-04	0.0151
2,5-Dihydroxybenzoic acid	DMSO	971	2.463	0.976	3.874	8.65E-04	0.0156
p-Tolualdehyde	DMSO	873	2.406	0.921	3.839	0.0012	0.0172
Lapachol	DMSO	811	2.354	0.861	3.925	0.0026	0.0177
Piperonyl alcohol	DMSO	1008	2.29	0.901	3.674	0.0012	0.0167
Sabinene	DMSO	823	2.166	0.574	3.714	0.0069	0.0198
Cadaverine	DMSO	788	2.056	0.42	3.664	0.013	0.0219
Sinomenine hydrochloride	DMSO	883	2.01	0.481	3.531	0.0098	0.0214
(-)-Cedrene	DMSO	1036	2.003	0.494	3.446	0.0078	0.0203
Pellitorine (p)	DMSO	846	1.956	0.431	3.553	0.014	0.0229
Piperine (p)	DMSO	787	1.949	0.41	3.518	0.014	0.0224
^beta-Citronellol	DMSO	860	1.904	0.47	3.345	0.0094	0.0208
Geranyl acetate	DMSO	890	1.807	0.275	3.329	0.0204	0.0245
Apigenin (p)	DMSO	754	1.761	0.114	3.355	0.0332	0.0266
Salsolidine	DMSO	748	1.706	0.113	3.244	0.0327	0.0255
4-Methoxybenzaldehyde	DMSO	901	1.652	0.205	3.117	0.0262	0.025
Nerolidol	DMSO	781	1.556	0.112	3.041	0.0373	0.0271
2-Nonylquinolin-4(1H)-one (p)	DMSO	803	1.511	0.051	2.933	0.0398	0.0276
Caffeine	DMSO	859	1.427	-0.098	2.869	0.0595	0.0281
Agmatine	DMSO	764	1.415	-0.229	2.992	0.0852	0.0297
Rotenone (p)	DMSO	865	1.405	-0.153	2.948	0.0757	0.0286
Menthol	DMSO	928	1.359	-0.159	2.899	0.0815	0.0292
^Citronellol	DMSO	767	1.28	-0.217	2.766	0.0927	0.0302
(+)-Carvone	DMSO	1052	1.122	-0.31	2.58	0.1279	0.0313
Spermidine	DMSO	977	0.904	-0.559	2.389	0.2292	0.0318
†Sitogluside (p)	DMSO	993	0.802	-0.787	2.277	0.3046	0.0349

Ajmalicine	DMSO	864	0.798	-0.724	2.329	0.3057	0.0354
Maslinic acid (p)	DMSO	879	0.791	-0.692	2.348	0.3075	0.0365
5-Aminolevulinic acid (hydrochloride)	DMSO	838	0.779	-0.658	2.288	0.3001	0.0344
Nootkatone	DMSO	963	0.671	-0.795	2.107	0.3645	0.0375
Alyssin	DMSO	970	0.601	-0.857	2.071	0.421	0.0396
Eucalyptol	DMSO	853	0.549	-1.033	2.06	0.4862	0.0406
Carnosic acid	DMSO	797	0.534	-1.109	2.127	0.5174	0.0411
Skatole	DMSO	978	0.436	-1.005	1.948	0.5629	0.0417
Water	DMSO	915	0.418	-1.043	1.889	0.5758	0.0422
trans-Anethole	DMSO	963	0.346	-1.117	1.851	0.6473	0.0432
Rosmarinic acid	DMSO	802	0.318	-1.193	1.89	0.6859	0.0448
Vincristine (sulfate)	DMSO	625	0.23	-1.497	1.953	0.7935	0.0458
Galanthamine	DMSO	886	0.203	-1.288	1.744	0.7931	0.0453
L-Citrulline	DMSO	883	0.129	-1.363	1.701	0.8688	0.0469
Salicylic acid	DMSO	1058	0.082	-1.329	1.516	0.9101	0.0474
Bergapten (p)	DMSO	806	0.079	-1.512	1.704	0.9231	0.0479
Deguelin (p)	DMSO	972	0.051	-1.425	1.597	0.9472	0.049
trans-Cinnamaldehyde	DMSO	879	0.043	-1.444	1.598	0.9555	0.0495
Curcumenol	DMSO	1045	-0.055	-1.488	1.424	0.9415	0.0484
Taurine	DMSO	985	-0.151	-1.61	1.341	0.841	0.0464
Cafestol	DMSO	1046	-0.31	-1.719	1.135	0.6699	0.0443
Pogostone	DMSO	856	-0.356	-1.886	1.211	0.6522	0.0438
Loganin	DMSO	928	-0.403	-1.956	1.024	0.5962	0.0427
Kaempferol	DMSO	999	-0.579	-2.004	0.854	0.4269	0.0401
L-Ornithine	DMSO	908	-0.61	-2.117	0.831	0.4174	0.0391
Anethole trithione (p)	DMSO	926	-0.628	-2.084	0.863	0.4035	0.0385
Carvacrol	DMSO	935	-0.65	-2.091	0.8	0.3781	0.038
Beta caryophyllene	DMSO	1041	-0.75	-2.169	0.597	0.2881	0.0333
Germacrene D	DMSO	1158	-0.754	-2.252	0.595	0.2992	0.0339
Micheliolide	DMSO	808	-0.762	-2.259	0.733	0.3184	0.037
(-)-Borneol	DMSO	867	-0.793	-2.327	0.716	0.3073	0.0359
Octyle acetate	DMSO	859	-0.875	-2.397	0.677	0.2646	0.0328
Myrcene	DMSO	769	-0.923	-2.505	0.62	0.2472	0.0323
p-Anisic acid	DMSO	1024	-1.168	-2.624	0.314	0.1191	0.0307
Oleanolic acid (p)	DMSO	730	-1.76	-3.362	-0.125	0.033	0.026
†Daucosterol (p)	DMSO	804	-1.885	-3.467	-0.321	0.0189	0.024
Ethyl p-methoxycinnamate	DMSO	751	-1.895	-3.396	-0.335	0.0152	0.0234
Methyl palmitate	DMSO	935	-1.978	-3.418	-0.567	0.0065	0.0193
Safranal	DMSO	848	-2.081	-3.542	-0.589	0.0057	0.0188
Ursolic acid (p)	DMSO	884	-2.229	-3.737	-0.737	0.0036	0.0182
Camphor	DMSO	941	-2.756	-4.166	-1.342	1.30E-04	0.0125
Spinosad (p)	DMSO	900	-2.797	-4.28	-1.379	1.57E-04	0.013
Salvinorin A propionate (p)	DMSO	926	-3.313	-4.793	-1.847	1.04E-05	0.0115
Ellagic acid (p)	DMSO	890	-3.744	-5.184	-2.379	1.68E-07	0.0089
2-Nonanone	DMSO	867	-4.576	-6.02	-3.097	8.42E-10	0.0068
Phytol	DMSO	625	-6.249	-7.8	-4.623	1.27E-14	0.0036
1-Octanol	DMSO	652	-7.446	-8.945	-5.917	5.39E-22	0.0031
Isoamyl alcohol	H2O	502	13.749	12.342	15.152	5.67E-82	0.0005
2-Methyl-1-butanol	H2O	381	11.294	9.483	13.039	1.41E-35	0.001

Thiophene	H2O	706	9.341	7.723	10.903	1.12E-30	0.0016
*2,3-Dihydrobenzofuran	H2O	705	8.776	7.138	10.356	1.12E-26	0.0021
Diacetyl	H2O	796	7.6	6.171	9.055	5.01E-25	0.0026
Phenylacetylene	H2O	496	6.032	4.136	7.734	4.99E-11	0.0042
Paeoniflorin	H2O	556	4.963	3.159	6.769	7.07E-08	0.0073
alpha-Phellandrene	H2O	777	4.949	3.271	6.52	2.34E-09	0.0052
Acetophenone	H2O	755	4.82	3.18	6.423	5.69E-09	0.0057
*Coumaran	H2O	754	4.512	2.942	6.024	9.54E-09	0.0063
Leonurine	H2O	766	4.115	2.436	5.682	6.67E-07	0.0089
Guaiazulene	H2O	958	3.886	2.422	5.379	2.60E-07	0.0078
L-Mimosine	H2O	916	3.864	2.354	5.385	5.83E-07	0.0083
Solasodine (p)	H2O	826	3.402	1.843	4.984	2.17E-05	0.0099
Isoquinoline	H2O	836	3.389	1.76	4.888	2.17E-05	0.0104
Furfural	H2O	796	3.201	1.56	4.793	1.04E-04	0.012
(-)-Huperzine A	H2O	911	2.96	1.392	4.435	1.38E-04	0.0125
Anisole	H2O	858	2.598	0.981	4.142	0.0013	0.0141
Limonin	H2O	907	2.404	0.847	3.964	0.0025	0.0151
Cinnamyl alcohol	H2O	857	2.395	0.771	3.941	0.0031	0.0156
Piperitenone	H2O	951	2.288	0.791	3.827	0.0031	0.0161
Ethyl palmitate	H2O	887	2.156	0.531	3.782	0.0093	0.0193
Carnosol	H2O	930	2.102	0.485	3.611	0.0084	0.0182
2,5-Dihydroxybenzoic acid	H2O	971	2.045	0.514	3.517	0.0076	0.0177
p-Tolualdehyde	H2O	873	1.987	0.397	3.483	0.0116	0.0203
Lapachol	H2O	811	1.936	0.308	3.582	0.0205	0.0208
Piperonyl alcohol	H2O	1008	1.872	0.43	3.297	0.0105	0.0198
Sabinene	H2O	823	1.747	0.129	3.39	0.0357	0.0214
Cadaverine	H2O	788	1.637	-0.068	3.252	0.0532	0.0234
Sinomenine hydrochloride	H2O	883	1.592	0.016	3.156	0.0469	0.0229
(-)-Cedrene	H2O	1036	1.584	-0.021	3.08	0.0452	0.0224
Pellitorine (p)	H2O	846	1.538	-0.211	3.091	0.0679	0.025
Piperine (p)	H2O	787	1.531	-0.104	3.112	0.0621	0.0245
^beta-Citronellol	H2O	860	1.486	-0.115	2.991	0.0608	0.024
Geranyl acetate	H2O	890	1.388	-0.257	2.948	0.0895	0.0255
Apigenin (p)	H2O	754	1.342	-0.304	3.018	0.1132	0.0271
Salsolidine	H2O	748	1.287	-0.364	2.899	0.122	0.0292
4-Methoxybenzaldehyde	H2O	901	1.233	-0.367	2.726	0.118	0.0281
Nerolidol	H2O	781	1.138	-0.413	2.689	0.1505	0.0307
2-Nonylquinolin-4(1H)-one (p)	H2O	803	1.093	-0.502	2.622	0.1704	0.0313
Caffeine	H2O	859	1.008	-0.528	2.521	0.1949	0.0339
Agmatine	H2O	764	0.996	-0.63	2.591	0.2254	0.0349
Rotenone (p)	H2O	865	0.986	-0.654	2.512	0.2219	0.0344
Menthol	H2O	928	0.941	-0.662	2.471	0.2392	0.0354
^Citronellol	H2O	767	0.861	-0.705	2.391	0.2755	0.0359
(+)-Carvone	H2O	1052	0.704	-0.733	2.151	0.3388	0.0375
Spermidine	H2O	977	0.486	-1.046	2.016	0.5339	0.0391
†Sitogluside (p)	H2O	993	0.384	-1.175	1.947	0.6297	0.0406
Ajmalicine	H2O	864	0.379	-1.246	1.947	0.6417	0.0417
Maslinic acid (p)	H2O	879	0.373	-1.208	1.963	0.6448	0.0427
5-Aminolevulinic acid (hydrochloride)	H2O	838	0.36	-1.164	1.883	0.6431	0.0422

Nootkatone	H2O	963	0.253	-1.31	1.695	0.7414	0.0453
Alyssin	H2O	970	0.183	-1.355	1.693	0.8141	0.0464
Eucalyptol	H2O	853	0.131	-1.52	1.711	0.8737	0.0474
Carnosic acid	H2O	797	0.116	-1.503	1.752	0.8888	0.0479
Skatole	H2O	978	0.017	-1.501	1.572	0.9822	0.0495
trans-Anethole	H2O	963	-0.072	-1.68	1.456	0.9282	0.049
Rosmarinic acid	H2O	802	-0.1	-1.654	1.485	0.9003	0.0484
Vincristine (sulfate)	H2O	625	-0.188	-1.963	1.556	0.8341	0.0469
Galanthamine	H2O	886	-0.216	-1.769	1.336	0.7856	0.0458
L-Citrulline	H2O	883	-0.289	-1.882	1.252	0.7175	0.0448
Salicylic acid	H2O	1058	-0.336	-1.861	1.087	0.6546	0.0438
Bergapten (p)	H2O	806	-0.339	-2.005	1.31	0.6883	0.0443
Deguelin (p)	H2O	972	-0.367	-1.87	1.199	0.639	0.0411
trans-Cinnamaldehyde	H2O	879	-0.375	-1.989	1.223	0.6472	0.0432
Curcumenol	H2O	1068	-0.473	-1.981	1.022	0.537	0.0396
Taurine	H2O	1045	-0.569	-2.095	1.017	0.4732	0.0385
Cafestol	H2O	985	-0.729	-2.238	0.679	0.3274	0.037
Pogostone	H2O	1046	-0.775	-2.391	0.809	0.3428	0.038
Loganin	H2O	856	-0.821	-2.41	0.684	0.2982	0.0365
Kaempferol	H2O	928	-0.998	-2.493	0.486	0.1894	0.0328
L-Ornithine	H2O	999	-1.028	-2.636	0.465	0.1937	0.0333
Anethole trithione (p)	H2O	908	-1.047	-2.616	0.412	0.1755	0.0323
Carvacrol	H2O	926	-1.068	-2.638	0.43	0.1723	0.0318
Beta caryophyllene	H2O	935	-1.168	-2.683	0.244	0.1178	0.0276
Germacrene D	H2O	1041	-1.172	-2.614	0.272	0.1113	0.0266
Micheliolide	H2O	1158	-1.18	-2.724	0.364	0.1341	0.0302
(-)-Borneol	H2O	808	-1.211	-2.782	0.362	0.1311	0.0297
Octyle acetate	H2O	867	-1.293	-2.994	0.255	0.1186	0.0286
Myrcene	H2O	859	-1.341	-2.95	0.272	0.1028	0.026
p-Anisic acid	H2O	769	-1.587	-3.143	-0.052	0.0442	0.0219
Oleanolic acid (p)	H2O	1024	-2.179	-3.833	-0.584	0.0086	0.0188
†Daucosterol (p)	H2O	730	-2.303	-3.945	-0.635	0.0064	0.0172
Ethyl p-methoxycinnamate	H2O	804	-2.314	-3.919	-0.7	0.0049	0.0167
Methyl palmitate	H2O	751	-2.397	-3.896	-0.89	0.0018	0.0146
Safranal	H2O	935	-2.5	-4.01	-1.003	0.0011	0.0135
Ursolic acid (p)	H2O	848	-2.648	-4.195	-1.07	8.98E-04	0.013
Camphor	H2O	884	-3.175	-4.717	-1.696	3.81E-05	0.0115
Spinosad (p)	H2O	941	-3.215	-4.792	-1.771	3.01E-05	0.0109
Salvinorin A propionate (p)	H2O	900	-3.732	-5.301	-2.233	1.86E-06	0.0094
Ellagic acid (p)	H2O	926	-4.162	-5.656	-2.786	1.31E-08	0.0068
2-Nonanone	H2O	890	-4.994	-6.571	-3.473	2.63E-10	0.0047
Phytol	H2O	867	-6.667	-8.326	-4.999	4.00E-15	0.0036
1-Octanol	H2O	625	-7.865	-9.412	-6.308	3.08E-23	0.0031
