# Supplementary material for: A high-throughput behavioral screening platform for measuring chemotaxis by C. elegans
Source: PLoS Biol. 2024 Jun 27;22(6):e3002672. doi: 10.1371/journal.pbio.3002672 (PMC11210793; doi:10.1371/journal.pbio.3002672)
Supplement: S3 Table — Tabulated list of the difference of the mean position for mutant worms between each test condition and a reference condition (aka “mean difference”), sample size (n = worms pooled across N = 3 biological replicates), 95% confidence intervals for the mean difference (5% CI, 95% CI). Control (DMSO) sample size was n = 851 for tax-4(p678), n = 936 for osm-9(ky10), and n = 915 tax-4(p678); osm-9(ky10) for all comparisons. Mean differences and confidence intervals obtained by bootstrapping using the Dabest statistical package [43]. These data are shown graphically in Fig 6 and are from assays conducted with tax-4(p678), osm-9(ky10), and tax-4(p678); osm-9(ky10) adult worms. (PDF) [file pbio.3002672.s005.pdf]

Test compound	Control	Test_n	Mean difference	CI_5	CI_95	Strain
(-)-Huperzine A	DMSO	675	2.507	1.196	3.777	PR678
1-octanol	DMSO	668	-3.953	-5.293	-2.707	PR678
2,3-Dihydrobenzofuran	DMSO	670	6.791	5.382	8.233	PR678
2,5-Dihydroxybenzoic acid	DMSO	998	1.257	0.015	2.37	PR678
2-Methyl-1-butanol	DMSO	640	1.723	0.325	3.059	PR678
2-nonanone	DMSO	767	-6.326	-7.514	-5.134	PR678
Acetophenone	DMSO	636	-4.155	-5.44	-2.91	PR678
Anisole	DMSO	777	6.443	5.207	7.7	PR678
Camphor	DMSO	624	1.511	0.138	2.831	PR678
Carnosol	DMSO	635	6.716	5.223	8.15	PR678
Cinnamyl alcohol	DMSO	727	4.824	3.422	6.117	PR678
Coumaran	DMSO	808	4.437	3.143	5.693	PR678
Daucosterol	DMSO	673	4.354	3.02	5.66	PR678
Diacetyl	DMSO	738	12.024	10.764	13.222	PR678
Ellagic acid	DMSO	671	3.74	2.417	5.034	PR678
Ethyl p-methoxycinnamate	DMSO	620	0.164	-1.115	1.435	PR678
Ethyl palmitate	DMSO	681	2.073	0.764	3.35	PR678
Furfural	DMSO	574	-1.76	-3.071	-0.422	PR678
Guaiazulene	DMSO	593	1.399	0.053	2.774	PR678
Water	DMSO	523	3.816	2.391	5.156	PR678
Isoamyl alcohol	DMSO	889	3.361	2.089	4.599	PR678
Isoquinoline	DMSO	582	1.29	0.012	2.51	PR678
L-Mimosine	DMSO	884	1.955	0.725	3.121	PR678
Lapachol	DMSO	640	3.359	2.046	4.713	PR678
Leonurine	DMSO	555	3.117	1.725	4.466	PR678
Limonin	DMSO	782	2.044	0.741	3.283	PR678
Methyl palmitate	DMSO	798	3.058	1.888	4.282	PR678
Oleanolic acid	DMSO	617	5.09	3.591	6.546	PR678
Paeoniflorin	DMSO	595	2.592	1.236	3.957	PR678
Phenylacetylene	DMSO	794	3.332	2.119	4.579	PR678
Phytol	DMSO	626	4.162	2.887	5.405	PR678
Piperitenone	DMSO	863	5.808	4.469	7.081	PR678
Piperonyl Alcohol	DMSO	615	2.855	1.46	4.164	PR678
Sabinene	DMSO	816	3.51	2.242	4.766	PR678
Salvinorin A Propionate	DMSO	714	2.695	1.401	3.977	PR678
Sinomenine hydrochloride	DMSO	673	3.462	2.183	4.745	PR678
Solasodine	DMSO	790	4.739	3.416	5.97	PR678
Spinosad	DMSO	846	2.191	0.973	3.418	PR678
Thiophene	DMSO	620	2.925	1.572	4.242	PR678
Ursolic acid	DMSO	559	3.597	2.163	4.994	PR678
p-Tolualdehyde	DMSO	734	-0.583	-1.815	0.656	PR678
alpha-Phellandrene	DMSO	879	2.643	1.416	3.875	PR678
(-)-Huperzine A	DMSO	976	0.283	-1.202	1.854	CX10

1-octanol	DMSO	806	-1.173	-2.655	0.347	CX10
2,3-Dihydrobenzofuran	DMSO	796	9.736	8.303	11.154	CX10
2,5-Dihydroxybenzoic acid	DMSO	840	-1.52	-3	0.088	CX10
2-Methyl-1-butanol	DMSO	558	10.784	9.256	12.363	CX10
2-nonanone	DMSO	861	-3.745	-5.229	-2.16	CX10
Acetophenone	DMSO	1056	1.139	-0.365	2.594	CX10
Anisole	DMSO	760	4.987	3.459	6.677	CX10
Camphor	DMSO	1049	1.568	0.083	3.055	CX10
Carnosol	DMSO	974	1.664	0.193	3.204	CX10
Cinnamyl alcohol	DMSO	1007	1.396	-0.085	2.902	CX10
Coumaran	DMSO	987	2.801	1.304	4.346	CX10
Daucosterol	DMSO	1002	0.196	-1.364	1.754	CX10
Diacetyl	DMSO	861	3.998	2.468	5.489	CX10
Ellagic acid	DMSO	1042	0.994	-0.526	2.439	CX10
Ethyl p-methoxycinnamate	DMSO	894	0.882	-0.538	2.276	CX10
Ethyl palmitate	DMSO	815	0.388	-1.19	1.952	CX10
Furfural	DMSO	780	3.522	1.92	5.09	CX10
Guaiazulene	DMSO	861	-2.027	-3.497	-0.525	CX10
Water	DMSO	909	2.071	0.554	3.541	CX10
Isoamyl alcohol	DMSO	711	11.387	9.916	12.882	CX10
Isoquinoline	DMSO	994	1.71	0.223	3.175	CX10
L-Mimosine	DMSO	855	0.684	-0.812	2.197	CX10
Lapachol	DMSO	1126	0.36	-1.049	1.817	CX10
Leonurine	DMSO	1061	-0.263	-1.726	1.199	CX10
Limonin	DMSO	973	2.249	0.792	3.709	CX10
Methyl palmitate	DMSO	952	1.587	0.087	3.159	CX10
Oleanolic acid	DMSO	908	-1.868	-3.371	-0.368	CX10
Paeoniflorin	DMSO	825	4.498	2.976	6.031	CX10
Phenylacetylene	DMSO	768	5.656	4.108	7.252	CX10
Phytol	DMSO	755	0.505	-1.089	2.096	CX10
Piperitenone	DMSO	875	4.663	3.171	6.127	CX10
Piperonyl Alcohol	DMSO	962	-4.272	-5.744	-2.766	CX10
Sabinene	DMSO	939	1.9	0.345	3.413	CX10
Salvinorin A Propionate	DMSO	964	-0.629	-2.084	1	CX10
Sinomenine hydrochloride	DMSO	1028	-1.037	-2.516	0.471	CX10
Solasodine	DMSO	1051	-0.543	-2.039	0.908	CX10
Spinosad	DMSO	895	0.993	-0.553	2.536	CX10
Thiophene	DMSO	897	1.565	-0.022	3.119	CX10
Ursolic acid	DMSO	869	0.112	-1.384	1.615	CX10
p-Tolualdehyde	DMSO	954	2.369	0.88	3.945	CX10
alpha-Phellandrene	DMSO	962	2.3	0.835	3.801	CX10
(-)-Huperzine A	DMSO	596	-0.435	-1.591	0.675	GN1077
1-octanol	DMSO	811	-0.041	-0.936	0.853	GN1077
2,3-Dihydrobenzofuran	DMSO	851	-1.425	-2.399	-0.482	GN1077

2,5-Dihydroxybenzoic acid	DMSO	1024	-0.758	-1.629	0.081	GN1077
2-Methyl-1-butanol	DMSO	434	-2.09	-3.444	-0.752	GN1077
2-nonanone	DMSO	709	-2.109	-3.044	-1.217	GN1077
Acetophenone	DMSO	830	-2.663	-3.556	-1.767	GN1077
Anisole	DMSO	668	-0.644	-1.665	0.35	GN1077
Camphor	DMSO	857	-0.214	-1.216	0.732	GN1077
Carnosol	DMSO	693	-0.379	-1.344	0.546	GN1077
Cinnamyl alcohol	DMSO	554	-0.529	-1.719	0.685	GN1077
Coumaran	DMSO	841	-1.106	-2.007	-0.226	GN1077
Daucosterol	DMSO	642	1.867	0.797	2.996	GN1077
Diacetyl	DMSO	836	-0.553	-1.417	0.351	GN1077
Ellagic acid	DMSO	887	-0.999	-1.885	-0.117	GN1077
Ethyl p-methoxycinnamate	DMSO	784	0.348	-0.571	1.248	GN1077
Ethyl palmitate	DMSO	961	-1.321	-2.299	-0.361	GN1077
Furfural	DMSO	733	-1.74	-2.628	-0.835	GN1077
Guaiazulene	DMSO	873	-0.761	-1.726	0.256	GN1077
Water	DMSO	784	-1.167	-2.06	-0.277	GN1077
Isoamyl alcohol	DMSO	703	-1.151	-2.043	-0.251	GN1077
Isoquinoline	DMSO	732	-1.789	-2.652	-0.943	GN1077
L-Mimosine	DMSO	700	0.141	-0.777	1.026	GN1077
Lapachol	DMSO	632	-1.912	-2.954	-0.914	GN1077
Leonurine	DMSO	619	-0.248	-1.239	0.691	GN1077
Limonin	DMSO	722	-0.283	-1.295	0.713	GN1077
Methyl palmitate	DMSO	656	-1.244	-2.16	-0.294	GN1077
Oleanolic acid	DMSO	675	-0.558	-1.557	0.442	GN1077
Paeoniflorin	DMSO	664	-1.207	-2.325	-0.14	GN1077
Phenylacetylene	DMSO	495	-1.36	-2.34	-0.331	GN1077
Phytol	DMSO	678	-1.793	-2.723	-0.88	GN1077
Piperitenone	DMSO	747	-0.641	-1.638	0.328	GN1077
Piperonyl Alcohol	DMSO	745	-1.812	-2.737	-0.91	GN1077
Sabinene	DMSO	681	-1.018	-2.02	0.006	GN1077
Salvinorin A Propionate	DMSO	714	-1.218	-2.163	-0.303	GN1077
Sinomenine hydrochloride	DMSO	897	-1.688	-2.576	-0.775	GN1077
Solasodine	DMSO	702	1.259	0.214	2.336	GN1077
Spinosad	DMSO	764	0.921	-0.017	1.928	GN1077
Thiophene	DMSO	861	-1.322	-2.201	-0.448	GN1077
Ursolic acid	DMSO	868	-1.521	-2.45	-0.639	GN1077
p-Tolualdehyde	DMSO	824	-0.262	-1.151	0.663	GN1077
alpha-Phellandrene	DMSO	764	-1.078	-2.012	-0.176	GN1077
