# Supplementary material for: A high-throughput behavioral screening platform for measuring chemotaxis by C. elegans
Source: PLoS Biol. 2024 Jun 27;22(6):e3002672. doi: 10.1371/journal.pbio.3002672 (PMC11210793; doi:10.1371/journal.pbio.3002672)
Supplement: S4 Table — Tabulated list of the |ΔΔ| values for each test condition and pairwise comparisons of the indicated strains (Strain1, Strain2), and 95% confidence intervals for the |ΔΔ|. The |ΔΔ| and confidence intervals were obtained by bootstrapping via the Dabest statistical package [43]. Strain [genotype]: N2 [wild-type], GN1077 [tax-4(pr678);osm-9(ky10)], CX10 [osm-9(ky10)], and PR678 [tax-4(p678)]. (PDF) [file pbio.3002672.s006.pdf]

Test compound	CI_5	CI_95	Delta-delta	Strain 1	Strain 2
(-)-Huperzine A	-5.646	-1.986	-3.813	GN1077	N2
1-octanol	5.703	9.104	7.406	GN1077	N2
2,3-Dihydrobenzofuran	-12.331	-8.728	-10.619	GN1077	N2
2,5-Dihydroxybenzoic acid	-4.863	-1.469	-3.221	GN1077	N2
2-Methyl-1-butanol	-15.918	-11.621	-13.802	GN1077	N2
2-nonanone	0.650	4.167	2.467	GN1077	N2
Acetophenone	-9.646	-6.084	-7.901	GN1077	N2
Anisole	-5.446	-1.800	-3.661	GN1077	N2
Camphor	0.892	4.240	2.542	GN1077	N2
Carnosol	-4.629	-1.139	-2.899	GN1077	N2
Cinnamyl alcohol	-5.274	-1.386	-3.342	GN1077	N2
Coumaran	-7.705	-4.306	-6.037	GN1077	N2
Daucosterol	1.864	5.701	3.751	GN1077	N2
Diacetyl	-10.197	-6.903	-8.572	GN1077	N2
Ellagic acid	1.123	4.446	2.745	GN1077	N2
Ethyl p-methoxycinnamate	0.497	4.095	2.243	GN1077	N2
Ethyl palmitate	-5.656	-2.052	-3.895	GN1077	N2
Furfural	-7.098	-3.556	-5.359	GN1077	N2
Guaiazulene	-6.765	-3.375	-5.065	GN1077	N2
Water	-3.296	0.172	-1.586	GN1077	N2
Isoamyl alcohol	-16.891	-13.635	-15.318	GN1077	N2
Isoquinoline	-7.372	-3.885	-5.597	GN1077	N2
L-Mimosine	-5.873	-2.464	-4.141	GN1077	N2
Lapachol	-6.107	-2.442	-4.266	GN1077	N2
Leonurine	-6.601	-2.893	-4.781	GN1077	N2
Limonin	-4.947	-1.293	-3.106	GN1077	N2
Methyl palmitate	-0.995	2.428	0.735	GN1077	N2
Oleanolic acid	-0.559	3.098	1.202	GN1077	N2
Paeoniflorin	-8.606	-4.582	-6.588	GN1077	N2
Phenylacetylene	-9.823	-5.740	-7.810	GN1077	N2
Phytol	2.564	6.239	4.456	GN1077	N2
Piperitenone	-5.037	-1.583	-3.348	GN1077	N2
Piperonyl alcohol	-5.721	-2.376	-4.103	GN1077	N2
Sabinene	-4.946	-1.221	-3.183	GN1077	N2
Salvinorin A propionate	0.379	3.847	2.095	GN1077	N2
Sinomenine hydrochloride	-5.387	-1.905	-3.698	GN1077	N2
Solasodine	-4.383	-0.653	-2.561	GN1077	N2
Spinosad	2.014	5.509	3.717	GN1077	N2
Thiophene	-12.779	-9.263	-11.082	GN1077	N2
Ursolic acid	-1.001	2.501	0.708	GN1077	N2
p-Tolualdehyde	-4.384	-0.951	-2.667	GN1077	N2
alpha-Phellandrene	-8.231	-4.663	-6.446	GN1077	N2
(-)-Huperzine A	-2.809	1.108	-0.871	PR678	N2

1-octanol	1.517	5.433	3.493	PR678	N2
2,3-Dihydrobenzofuran	-4.525	-0.344	-2.403	PR678	N2
2,5-Dihydroxybenzoic acid	-3.010	0.670	-1.206	PR678	N2
2-Methyl-1-butanol	-12.184	-7.739	-9.990	PR678	N2
2-nonanone	-3.715	0.142	-1.750	PR678	N2
Acetophenone	-11.459	-7.467	-9.394	PR678	N2
Anisole	1.402	5.390	3.427	PR678	N2
Camphor	2.323	6.192	4.268	PR678	N2
Carnosol	2.075	6.238	4.195	PR678	N2
Cinnamyl alcohol	-0.030	4.002	2.010	PR678	N2
Coumaran	-2.388	1.468	-0.494	PR678	N2
Daucosterol	4.115	8.281	6.238	PR678	N2
Diacetyl	2.148	5.876	4.005	PR678	N2
Ellagic acid	5.569	9.359	7.484	PR678	N2
Ethyl p-methoxycinnamate	0.070	4.004	2.059	PR678	N2
Ethyl palmitate	-2.587	1.507	-0.502	PR678	N2
Furfural	-7.417	-3.388	-5.379	PR678	N2
Guaiazulene	-4.775	-0.936	-2.906	PR678	N2
Water	1.276	5.399	3.397	PR678	N2
Isoamyl alcohol	-12.602	-8.954	-10.806	PR678	N2
Isoquinoline	-4.569	-0.603	-2.518	PR678	N2
L-Mimosine	-4.180	-0.435	-2.327	PR678	N2
Lapachol	-1.057	3.038	1.005	PR678	N2
Leonurine	-3.443	0.623	-1.417	PR678	N2
Limonin	-2.736	1.142	-0.779	PR678	N2
Methyl palmitate	3.110	6.904	5.036	PR678	N2
Oleanolic acid	4.736	9.125	6.850	PR678	N2
Paeoniflorin	-4.926	-0.601	-2.790	PR678	N2
Phenylacetylene	-5.255	-0.906	-3.118	PR678	N2
Phytol	8.329	12.420	10.411	PR678	N2
Piperitenone	1.115	4.972	3.102	PR678	N2
Piperonyl alcohol	-1.424	2.442	0.564	PR678	N2
Sabinene	-0.597	3.329	1.345	PR678	N2
Salvinorin A propionate	4.027	7.908	6.008	PR678	N2
Sinomenine hydrochloride	-0.508	3.345	1.451	PR678	N2
Solasodine	-1.131	2.879	0.919	PR678	N2
Spinosad	3.095	6.850	4.988	PR678	N2
Thiophene	-8.857	-4.802	-6.835	PR678	N2
Ursolic acid	3.812	7.798	5.826	PR678	N2
p-Tolualdehyde	-4.845	-1.121	-2.989	PR678	N2
alpha-Phellandrene	-4.737	-0.741	-2.724	PR678	N2
(-)-Huperzine A	-5.213	-1.065	-3.095	CX10	N2
1-octanol	4.203	8.333	6.273	CX10	N2
2,3-Dihydrobenzofuran	-1.635	2.671	0.541	CX10	N2

2,5-Dihydroxybenzoic acid	-6.129	-1.903	-3.983	CX10	N2
2-Methyl-1-butanol	-3.197	1.362	-0.928	CX10	N2
2-nonanone	-1.315	3.008	0.831	CX10	N2
Acetophenone	-6.177	-2.017	-4.099	CX10	N2
Anisole	-0.221	4.231	1.970	CX10	N2
Camphor	2.280	6.359	4.324	CX10	N2
Carnosol	-3.022	1.225	-0.856	CX10	N2
Cinnamyl alcohol	-3.580	0.644	-1.418	CX10	N2
Coumaran	-4.180	-0.009	-2.130	CX10	N2
Daucosterol	-0.144	4.308	2.080	CX10	N2
Diacetyl	-6.107	-1.996	-4.021	CX10	N2
Ellagic acid	2.735	6.760	4.738	CX10	N2
Ethyl p-methoxycinnamate	0.685	4.852	2.777	CX10	N2
Ethyl palmitate	-4.409	-0.031	-2.186	CX10	N2
Furfural	-2.271	2.250	-0.098	CX10	N2
Guaiazulene	-8.349	-4.327	-6.332	CX10	N2
Water	-0.388	3.812	1.653	CX10	N2
Isoamyl alcohol	-4.732	-0.853	-2.780	CX10	N2
Isoquinoline	-4.141	0.103	-2.097	CX10	N2
L-Mimosine	-5.719	-1.428	-3.598	CX10	N2
Lapachol	-4.117	0.182	-1.994	CX10	N2
Leonurine	-6.955	-2.730	-4.797	CX10	N2
Limonin	-2.688	1.552	-0.574	CX10	N2
Methyl palmitate	1.487	5.699	3.565	CX10	N2
Oleanolic acid	-2.342	2.020	-0.108	CX10	N2
Paeoniflorin	-3.276	1.384	-0.883	CX10	N2
Phenylacetylene	-3.213	1.545	-0.794	CX10	N2
Phytol	4.498	9.074	6.754	CX10	N2
Piperitenone	-0.086	3.927	1.956	CX10	N2
Piperonyl alcohol	-8.682	-4.569	-6.562	CX10	N2
Sabinene	-2.479	1.883	-0.265	CX10	N2
Salvinorin A propionate	0.553	4.783	2.685	CX10	N2
Sinomenine hydrochloride	-5.159	-0.922	-3.047	CX10	N2
Solasodine	-6.434	-2.189	-4.364	CX10	N2
Spinosad	1.710	5.986	3.790	CX10	N2
Thiophene	-10.251	-5.991	-8.195	CX10	N2
Ursolic acid	0.150	4.442	2.341	CX10	N2
p-Tolualdehyde	-2.042	2.114	-0.036	CX10	N2
alpha-Phellandrene	-5.219	-1.024	-3.068	CX10	N2
(-)-Huperzine A	-4.772	-1.283	-2.941	PR678	GN1077
1-octanol	2.371	5.513	3.912	PR678	GN1077
2,3-Dihydrobenzofuran	-9.946	-6.539	-8.216	PR678	GN1077
2,5-Dihydroxybenzoic acid	-3.459	-0.520	-2.015	PR678	GN1077
2-Methyl-1-butanol	-5.712	-1.911	-3.813	PR678	GN1077

2-nonanone	2.761	5.709	4.217	PR678	GN1077
Acetophenone	-0.111	3.047	1.492	PR678	GN1077
Anisole	-8.630	-5.376	-7.088	PR678	GN1077
Camphor	-3.367	0.009	-1.725	PR678	GN1077
Carnosol	-8.770	-5.264	-7.094	PR678	GN1077
Cinnamyl alcohol	-7.148	-3.597	-5.352	PR678	GN1077
Coumaran	-7.063	-3.995	-5.543	PR678	GN1077
Daucosterol	-4.257	-0.676	-2.487	PR678	GN1077
Diacetyl	-14.111	-11.054	-12.577	PR678	GN1077
Ellagic acid	-6.234	-3.106	-4.739	PR678	GN1077
Ethyl p-methoxycinnamate	-1.374	1.684	0.184	PR678	GN1077
Ethyl palmitate	-5.069	-1.838	-3.393	PR678	GN1077
Furfural	-1.545	1.674	0.020	PR678	GN1077
Guaiazulene	-3.798	-0.470	-2.159	PR678	GN1077
Water	-6.680	-3.359	-4.983	PR678	GN1077
Isoamyl alcohol	-6.042	-2.955	-4.512	PR678	GN1077
Isoquinoline	-4.604	-1.533	-3.079	PR678	GN1077
L-Mimosine	-3.361	-0.275	-1.814	PR678	GN1077
Lapachol	-6.917	-3.553	-5.271	PR678	GN1077
Leonurine	-5.000	-1.681	-3.364	PR678	GN1077
Limonin	-3.985	-0.714	-2.327	PR678	GN1077
Methyl palmitate	-5.803	-2.732	-4.301	PR678	GN1077
Oleanolic acid	-7.381	-3.858	-5.648	PR678	GN1077
Paeoniflorin	-5.495	-2.068	-3.799	PR678	GN1077
Phenylacetylene	-6.283	-3.075	-4.692	PR678	GN1077
Phytol	-7.554	-4.319	-5.955	PR678	GN1077
Piperitenone	-8.009	-4.797	-6.449	PR678	GN1077
Piperonyl alcohol	-6.288	-2.974	-4.667	PR678	GN1077
Sabinene	-6.194	-2.885	-4.528	PR678	GN1077
Salvinorin A propionate	-5.504	-2.305	-3.913	PR678	GN1077
Sinomenine hydrochloride	-6.658	-3.583	-5.149	PR678	GN1077
Solasodine	-5.099	-1.824	-3.480	PR678	GN1077
Spinosad	-2.824	0.308	-1.271	PR678	GN1077
Thiophene	-5.878	-2.674	-4.247	PR678	GN1077
Ursolic acid	-6.774	-3.432	-5.118	PR678	GN1077
p-Tolualdehyde	-1.161	1.777	0.322	PR678	GN1077
alpha-Phellandrene	-5.241	-2.219	-3.721	PR678	GN1077
(-)-Huperzine A	-2.618	1.119	-0.717	CX10	GN1077
1-octanol	-0.646	2.898	1.133	CX10	GN1077
2,3-Dihydrobenzofuran	-12.855	-9.444	-11.160	CX10	GN1077
2,5-Dihydroxybenzoic acid	-0.970	2.539	0.762	CX10	GN1077
2-Methyl-1-butanol	-14.822	-10.784	-12.874	CX10	GN1077
2-nonanone	-0.170	3.413	1.635	CX10	GN1077
Acetophenone	-5.537	-2.078	-3.802	CX10	GN1077

Anisole	-7.440	-3.651	-5.631	CX10	GN1077
Camphor	-3.642	-0.042	-1.782	CX10	GN1077
Carnosol	-3.848	-0.309	-2.043	CX10	GN1077
Cinnamyl alcohol	-3.895	0.026	-1.925	CX10	GN1077
Coumaran	-5.670	-2.195	-3.907	CX10	GN1077
Daucosterol	-0.218	3.641	1.671	CX10	GN1077
Diacetyl	-6.293	-2.774	-4.551	CX10	GN1077
Ellagic acid	-3.698	-0.271	-1.993	CX10	GN1077
Ethyl p-methoxycinnamate	-2.211	1.210	-0.534	CX10	GN1077
Ethyl palmitate	-3.598	0.060	-1.709	CX10	GN1077
Furfural	-7.132	-3.403	-5.261	CX10	GN1077
Guaiazulene	-0.554	3.086	1.267	CX10	GN1077
Water	-4.998	-1.528	-3.238	CX10	GN1077
Isoamyl alcohol	-14.353	-10.877	-12.539	CX10	GN1077
Isoquinoline	-5.278	-1.825	-3.500	CX10	GN1077
L-Mimosine	-2.437	1.233	-0.543	CX10	GN1077
Lapachol	-4.096	-0.489	-2.272	CX10	GN1077
Leonurine	-1.738	1.729	0.016	CX10	GN1077
Limonin	-4.352	-0.673	-2.533	CX10	GN1077
Methyl palmitate	-4.686	-1.065	-2.830	CX10	GN1077
Oleanolic acid	-0.549	3.213	1.310	CX10	GN1077
Paeoniflorin	-7.549	-3.719	-5.705	CX10	GN1077
Phenylacetylene	-8.885	-5.164	-7.016	CX10	GN1077
Phytol	-4.060	-0.439	-2.298	CX10	GN1077
Piperitenone	-7.161	-3.527	-5.304	CX10	GN1077
Piperonyl alcohol	0.671	4.304	2.460	CX10	GN1077
Sabinene	-4.740	-1.101	-2.918	CX10	GN1077
Salvinorin A propionate	-2.434	1.121	-0.589	CX10	GN1077
Sinomenine hydrochloride	-2.388	1.127	-0.651	CX10	GN1077
Solasodine	-0.057	3.643	1.802	CX10	GN1077
Spinosad	-1.881	1.756	-0.073	CX10	GN1077
Thiophene	-4.654	-1.154	-2.887	CX10	GN1077
Ursolic acid	-3.456	0.124	-1.633	CX10	GN1077
p-Tolualdehyde	-4.475	-0.856	-2.631	CX10	GN1077
alpha-Phellandrene	-5.144	-1.670	-3.378	CX10	GN1077
(-)-Huperzine A	0.220	4.187	2.224	PR678	CX10
1-octanol	-4.800	-0.794	-2.780	PR678	CX10
2,3-Dihydrobenzofuran	-4.904	-0.886	-2.945	PR678	CX10
2,5-Dihydroxybenzoic acid	0.804	4.744	2.777	PR678	CX10
2-Methyl-1-butanol	-11.208	-7.054	-9.061	PR678	CX10
2-nonanone	-4.520	-0.652	-2.581	PR678	CX10
Acetophenone	-7.218	-3.342	-5.294	PR678	CX10
Anisole	-0.673	3.477	1.456	PR678	CX10
Camphor	-2.101	1.970	-0.057	PR678	CX10

Carnosol	2.982	7.124	5.051	PR678	CX10
Cinnamyl alcohol	1.390	5.459	3.427	PR678	CX10
Coumaran	-0.321	3.635	1.636	PR678	CX10
Daucosterol	2.109	6.191	4.158	PR678	CX10
Diacetyl	6.081	10.037	8.026	PR678	CX10
Ellagic acid	0.781	4.800	2.746	PR678	CX10
Ethyl p-methoxycinnamate	-2.571	1.156	-0.717	PR678	CX10
Ethyl palmitate	-0.395	3.780	1.685	PR678	CX10
Furfural	-7.343	-3.180	-5.281	PR678	CX10
Guaiazulene	1.403	5.540	3.426	PR678	CX10
Water	-0.347	3.850	1.744	PR678	CX10
Isoamyl alcohol	-9.929	-6.063	-8.026	PR678	CX10
Isoquinoline	-2.388	1.458	-0.420	PR678	CX10
L-Mimosine	-0.761	3.221	1.271	PR678	CX10
Lapachol	1.013	4.985	2.999	PR678	CX10
Leonurine	1.325	5.447	3.380	PR678	CX10
Limonin	-2.115	1.766	-0.205	PR678	CX10
Methyl palmitate	-0.473	3.347	1.471	PR678	CX10
Oleanolic acid	4.797	8.986	6.958	PR678	CX10
Paeoniflorin	-3.982	0.135	-1.906	PR678	CX10
Phenylacetylene	-4.399	-0.214	-2.324	PR678	CX10
Phytol	1.591	5.681	3.657	PR678	CX10
Piperitenone	-0.810	3.189	1.145	PR678	CX10
Piperonyl alcohol	5.147	9.211	7.126	PR678	CX10
Sabinene	-0.340	3.541	1.610	PR678	CX10
Salvinorin A propionate	1.233	5.282	3.323	PR678	CX10
Sinomenine hydrochloride	2.538	6.469	4.499	PR678	CX10
Solasodine	3.299	7.206	5.282	PR678	CX10
Spinosad	-0.799	3.237	1.198	PR678	CX10
Thiophene	-0.665	3.438	1.360	PR678	CX10
Ursolic acid	1.355	5.499	3.485	PR678	CX10
p-Tolualdehyde	-4.820	-0.908	-2.953	PR678	CX10
alpha-Phellandrene	-1.586	2.268	0.343	PR678	CX10
