# Supplementary material for: A high-throughput behavioral screening platform for measuring chemotaxis by C. elegans
Source: PLoS Biol. 2024 Jun 27;22(6):e3002672. doi: 10.1371/journal.pbio.3002672 (PMC11210793; doi:10.1371/journal.pbio.3002672)
Supplement: Attachment — Submitted filename: Fryer Response2Reviewers.docx [file pbio.3002672.s011.docx]

# Response to Reviewers

We thank all three reviewers for their evaluation of our initial submission and appreciation of the value of the platform we presented. We particularly appreciate the suggestions for improvement of the presentation of the method, statistical analysis, and consideration of limitations.

We thoroughly revised our statistical approach to rely primarily on bootstrapping and estimation statistics and revised the text to assist readers more familiar with classical null hypothesis significance testing in digesting this alternative approach. We also implemented a 2x2 factorial design to quantitatively evaluate how the mutants we tested affected the measured chemotaxis responses, yielding ΔΔ values for each mutant against wild-type and for each single mutant against the double mutant (*tax-4;osm-9*).

This revision includes significant revisions or additions to the following figures panels (all figures are re-formatted to according to the journal’s instructions):

1. Fig 1B, new diagram showing thickness of solid media and foam inserts
2. Fig 2D, new analyses showing mean positions for each technical replicate
3. Fig 2E, new analyses comparing differences of the means derived by averaging replicates and those derived by pooling the positions of individual animals across replicates.
4. Fig 5, new annotations indicating nominally identical SMs (brown text), those identified as false positives following B-H correction for multiple tests.
5. Fig 6A, new analyses comparing the chemotaxis response in wild-type and mutant animals.
6. Fig 6B, new 3-D plot of SMs with symbols encoded according to valence (color) and response strength (size)
7. Fig 7, new analyses based on the wild-type vs. mutant comparison shown in Fig 6 and a parallel analysis comparing single *tax-4* and *osm-9* mutants to *tax-4;osm-9* double mutants.
8. S1 Figure, two images showing: 1) foam seals in assay plate lids and 2) flatbed scanner in black box
9. S1 Table, new annotations of SMs showing visual evidence of precipitates
10. S2 Table, table of the data shown in Fig 5 including the total number of worms analyzed across three biological replicates.
11. S3 Table, parallel to S2 Table but showing the data for *tax-4, osm-9*, and *tax-4;osm-9* mutants displayed in Fig 6.
12. S4 Table, table of the data underpinning the classification scheme used in Fig 7

A point-by-point response is provided below, with reviewers’ critiques in plain, black text and authors’ responses in *blue, italic text*.

### **Reviewer #1**:

Fryer et al. present a powerful new quantitative platform for measuring chemotaxis in *C. elegans*. This platform was optimized using a series of experimental and computational techniques that also combine creative custom-fabricated solutions. This approach was impressive and presents a method (and experimental paradigm) that other labs can mimic and follow. The use of open-source tools and python were laudable. Beyond the technique, the authors test 90 different plant-derived small molecules for chemotaxis and find that ~40% elicit effects on the C. elegans N2 strain. Their results also have implications for how chemosensory neurons recognize compounds and elicit organismal behaviors (attraction or repulsion). I have only minor points and some specific edits to address. The manuscript is fun and impressive.

*We thank the reviewer for their positive evaluation, appreciation, and feedback.*

#### Minor points:

(1) *C. elegans* is not a soil nematode. It is found in rotting fruits, flowers, tubers, etc. A variety of citations from the Felix and Andersen labs have shown this focus, so the two citations, which do show global distribution, should be amended or others added. Also, the section on page 11 dealing with the soil matrix should be edited. It is not clear from any study that they are found in the soil any more than the substrate on top of the soil.

*RESPONSE: We thank the reviewer for bringing this to our attention.*

*REVISION: To reflect findings in other studies, we modified the introduction to include a more precise discussion of the distribution of C. elegans in the wild (lines 95-97).*

(2) In the Methods, the authors state "independent biological replicates", but these words can mean many different things to different researchers. Please add specificity about the growth, bleach, synchronization, etc. independence of each sample. Additionally, pooling across all three replicates will conflate effects of the preparation technique. Please perform an ANOVA to measure the variance from the different preparation parameters in the experimental set up.

*RESPONSE: We thank the reviewer for these comments, which prompted us to compare the results obtained from individual replicates and those obtained by pooling the data across replicates. To do this, we took advantage of the experiments for which we have 16 technical replicates (Figure 2). In brief, we found no evidence for systematic variation among replicates, a finding which suggests that an ANOVA would not be informative.*

*REVISION: In the methods subsection “Chemotaxis assays”, we added the following sentence to clarify how our team defined independent biological replicates:“For each strain, we collected and analyzed the data of at least three biological replicates, which consisted of independently prepared age-synchronized worms tested on different days.” (lines 188-190). To assess if it was appropriate to pool our data, we compared the mean difference distributions derived by analyzing individual replicates and by pooling across them from the data represented in Fig 2D (chemoactive controls and null conditions). Overall, we found that these two approaches generated average values that are indistinguishable from one another, with the exception that pooling tightens the confidence intervals. These findings are summarized in the results sub-section “Platform performance and validation” and illustrated in Fig 2D. In addition, in Fig 2D, we represented the mean worm position for each replicate as a black circle.*

(3) Like most chemotaxis index calculations, animals near the starting zone are excluded from the analysis. How big of a region was excluded around the band of the starting position?

*RESPONSE: We thank the reviewer for this question.*

*REVISION: The central one-ninth of the arena is excluded for the chemotaxis index calculation. To address this question, in the methods sub-section “Data and Statistical Analysis”, we clarified how our software OWL computes a chemotaxis index value for each assay (lines 398-404).*

#### Specific edits:

(1) Page 11, "evolution-inspired approach" is not obvious to me. As a process or approach, use of the hypothesized natural interactions is not evolution. Perhaps natural environment is more appropriate.

*Upon reflection, we agree that the intended meaning was not clear. We have revised the text to read (lines 108-112): “Although this platform is compatible with any chemical library, we opted to screen plant SMs for their ability to evoke C. elegans chemotaxis. This choice is inspired by the interaction between plants and nematodes in natural environments and the idea that such a coevolution-inspired approach can deepen understanding of inter-species chemical cues and animal behavior.”*

(2) Page 19, the equation is not rendered correctly in the Methods PDF and could not be evaluated.

*Thank you for letting us know. As far as we can tell, this seems to be an issue with file conversion. To avoid this, we have reformatted the equation to be in-line with the text (line 380). We also added text to the Methods section to clarify how the chemotaxis index was calculated and that following established practice, we omitted animals remaining in the starting zone from the calculation.*

(3) Throughout, the figure insertion locations were left accidently as notes in the manuscript text.

*The figure insertions were retained as a guide to assist the reader in integrating the text and figures, but it seems that it was more of a distraction than an aid. We apologize and have deleted these notes.*

(4) Page 31, "were disrupted in both single of the mutants" should be "in both single mutants"

*Thank you. This has been corrected (line 890).*

(5) Page 31, "This findings indicate" should be "These"

*Thank you. With the new statistical analysis, this section has been revised and the former sentence with the error has been deleted.*

### **Reviewer #2**:

Fryer et al present a new accessible high throughput method for screening the valence of C. elegans chemotaxis to large libraries of small molecules. The approach presents advances in terms of the integration of liquid handling, plate-reading and computer-vision and does so in a way that should allow other groups to replicate the work. The novel findings, although not strictly required for a methods and resource format, are nonetheless interesting and clearly demonstrate the utility of the types of screens allowed by this approach. Therefore the work will be a valuable addition to the community.

*Thank you for the appreciation of the approach and especially for noting that this presentation should allow other groups to replicate the method, which was a very important goal for our research. We also appreciate the comment regarding the novel findings that are incorporated into the manuscript.*

In general, the work is well written, detailed and thorough, but there are a few blind spots that are important to address. I have provided detailed comments, but am still excited by the work and think that the concerns can be addressed with some clarification to the text, additional statistics, and a few straightforward control measurements.

*Thank you for the close reading and suggestions for improvement.*

#### Clarification of methods

Design of assay plates: the z-depth of the plates and thickness of gel gum are not shown. Specify whether the plates are enclosed, or whether there are air routes form one chamber to another. If the chambers are not sealed, then evaporation can deplete the concentration in the chamber in a molecule dependent manner (or travel to other chambers) which may confound the worm's response, so this is important to convey to the reader.

*We thank the reviewer for asking clarifying questions relating to our methods. As noted in detail below, we have added a diagram to Fig 1B showing the thickness of the solid media and the depth of the wells. We sealed air routes between chambers by adding a foam sheet to the plate lid, which we neglected to discuss in our initial submission. Revisions to the manuscript are summarized below:*

1. *To Fig 1B, we added a side view of the assay lane showing the total height (z-depth) and gel thickness.*
2. *In the methods sub-section titled “Chemical gradient set-up”, we added text noting that the 4-well plates used in our experiments are vented (line 240) and thus, to avoid cross-talk between lanes, we enclosed the vent by inserting a sheet of foam into the lid (lines 242-244). Additionally, an image of the set-up has been added to S1 Fig.*

#### Potential confounds

##### Odor cross contamination. A control experiment is needed to show that chemotaxis in one plate is not influenced by the introduction of an oderant into a neighboring assay plate. This is especially important for volatile compounds that evaporate easily. For this control experiment, the authors should choose an odorant from their library that is among the more volatile. Similarly, residue from one set of plates could remain in the instrument across experiments. Discussion is needed of steps taken to mitigate this concern. ‘

*RESPONSE: In our preliminary design work, we worried about cross-talk between adjacent lanes within each assay plate. We also found experimental data suggestive of cross-talk within a four-lane assay plate and conducted experiments to determine the size of this confound. Having found that it was significant, especially in the case of stronger attractants and repellents, we sought experimental strategies to reduce or eliminate this effect. Indeed, we found that such cross-talk was suppressed and nearly eliminated by inserting a sheet of EVA foam in the lid of the assay plate. This maneuver also seals each chamber, reducing evaporation noted above as a potential confound, as well as minimizing the chances of ‘residue’ lingering in the assay chamber.*

*REVISION: Prompted by the reviewer, we added text describing the use of foam sheets in the lid (lines 242-244) and added a panel showing the foam sheets to Figure S1. We also conducted additional experiments to determine if foam sheets are effective at suppressing cross-talk between lanes. In this image, you can readily see that for volatile odorants (isoamyl alcohol) the neighboring DMSO:DMSO arena (top well) is neutrally distributed* ***with*** *the lid insert (left), but heavily skewed toward the compound side* ***without*** *the lid insert (right). The less volatile odorant (1-octanol) does not obviously influence the animals in the neighboring assay lane whether or not the lid insert is present. These new observations are consistent with observations early in our design-build-test cycles, prompting us to begin using the lid inserts. All experiments in this manuscript were conducted with foam lid inserts in place during gradient establishment, with brief lid removal for addition of the worms to the arena, and replacement for the duration of chemotaxis.*

##### Relatedly, each small molecule likely has a different diffusion coefficient, solubility in the solvent, and absorption rate into the gum, which could impact the amount of small molecules experienced by the animal. For example, 2-nonanone, used in the library has been reported to have strong interactions with the substrate and walls: Akiko et. al., (2018). Measuring Spatiotemporal Dynamics of Odor Gradient for Small Animals by Gas Chromatography, Bio-protocol 8 (7): e2797. DOI: 10.21769/BioProtoc.2797 If the authors have an estimate for these properties they should provide them in a table. They should also comment in the text on how these properties may introduce potential confounds.

*RESPONSE: The reviewer is correct that our test compounds differ in their diffusion coefficients, solubility in solvent (DMSO), and volatility. They may also differ with respect to their partition into solid media (whether gellan gum or agar) or other physico-chemical properties. With respect to solvent solubility, our library included only compounds known to be soluble in DMSO, a solvent that is widely used in chemical screens done in academia and industry. That said, we might underestimate sensitivity to compounds with low partition coefficients, diffusion coefficients, low solubility, or low volatility. For these reasons, we cannot exclude the possibility that SMs that did not appear to induce either attraction or repulsion might be chemoactive under other conditions.*

*REVISION: In taking these comments into account, we expanded our methods sub-section titled “Chemical gradient set-up”(lines 242-244) and included a limitations section addressing the consequences of this limitation (lines 983-992). A subset of compounds formed visible precipitates in assays, an observation that points toward lower solubility for those compounds. They are denoted with (p)* *in S1 Table. These compounds were neither depleted nor enriched in the subset of chemoactive SMs, indicating that variations in DMSO solubility did not skew our screening results (lines 873-878).*

##### Temperature. Consumer flatbed scanners shine intense light that heat the sample in a non-uniform way during imaging. On some scanners the light "warms up" at one end of the instrument before scanning. It appears that in this method the entire scanner is also set into an enclosed box that likely accumulates heat over the course of experiments. Because worms thermotax and are exquisite thermosensors, it is important to measure and report the spatial or temporal thermal landscapes produced by the instrument.

- Temperature should be characterized at multiple locations within an assay plate during a typical set of experiments
- Spatiotemporal changes especially need to be measured during the 2 min scanning process
- Temperature should also be measured across multiple scans to see how temperature fluctuates, which could introduce across-plate confounds.

*RESPONSE: Having previously studied C. elegans thermotaxis and sensory thermotransduction (Ramot et al, J Neurosci 2008; Ramot et al, Nat Neurosci, 2008), we are keenly aware of this animal’s extraordinary sensitivity to temperature and thank the reviewer for bringing this concern to our attention. We have made the requested measurements, which are detailed below.*

*Before proceeding, we wish to correct a misinterpretation. Assay plates were scanned only once and they are not scanned repeatedly. Thus, for heating to affect our measurements, it must occur during a single scan.*

*During the 1-hour assay time, the plates are held in a dry cabinet maintained at 30% RH. At the end of the assay time, the plates are removed from the dry cabinet and placed, four at a time, on the surface of the flatbed scanner and all four plates are scanned in a single pass of the flatbed scanner (illustrated in S2 Figure).*

*We measured the temperature on the surface of the gellan gum during a single scan without the enclosure and during successive scans with the enclosure. Without the enclosure, we measured temperature with an infrared thermometer (Mastercool Inc., Model # 52225-A, ±0.1°C sensitivity, 2% accuracy) on the gellan gum surface (at four scanning locations) within the assay arenas before and during scans. We found that temperature was stable during scanning at four scanning locations (see ‘Surface Temperature During a Single Scan’, showing temperatures before (left) and after (right) scanning).*

*To identify whether there was any change in the ambient temperature of the enclosed scanning platform, we placed a LabJack Digit-TLH temperature logging device at the center of the scanning surface; the data logger was set to capture temperatures every 30 seconds. We covered the scanner and conducted 6 successive scans (2’15”) without lifting the cover. The temperature inside the chamber was 22.06°C prior to scanning and an average of 21.85±0.03°C (mean±sd, n=4) during the first scan (actual conditions) and did not change significantly during 3 scans (see “Effect of successive scans on temperature”). The temperature did rise to 22.74±0.08°C at the conclusion of the 6th and final scan. This corresponds to an increase of less than 1°C over ~15 minutes and the temperature is far below those known to be noxious for C. elegans (Glauser et al, Genetics, 2011).*

*Collectively, these measurements demonstrate that the single scans we used to image assay arenas produce no significant heat, even within a closed system the temperature is well within acceptable physiological limits.*

*REVISION: We revised the text in the Methods to inform readers that each assay plate was scanned only once and that the temperature in the scanning environment did not change during the single scan (lines 299-302).*

Statistics

The authors describe how one advantage of their approach is that it increases statistical power because it reports the individual location of each worm across plates. This allows for analysis based on the much larger number of worms (one dot per worm) rather than the number of assay plates (one dot per plate). But this approach implies that each worm is the fundamental independent measurement, and more justification is needed for that assumption. For example, there could be systematic biases shared by each assay plate: e.g. variability in the size of the small molecule droplet. One way to show that these systematics are small is to show, for a given condition, how the average worm location varies across plates (one dot per plate). Such a plot would also be valuable generally to show reproducibility across trials.

*RESPONSE: We thank the reviewer for these queries and comments, which have helped us to improve communication of our analytical and statistical approach. We added additional analyses similar to those suggested by the reviewer to show readers average worm location per assay lane alongside the results for the pooled data (described below).*

*There seem a few overlapping issues raised by the reviewer, which we endeavor to address here and in revisions to the manuscript:*

1. *Treating individual worms as the fundamental independent measurement.*
2. *Systematic variability in assay conditions*

*Treating individual worms as the fundamental independent measurement*

*Prior work has demonstrated that analyzing many individual worms is similar to the response found by testing a group of worms (Troemel, Kimmel & Bargmann, 1997). These findings imply that each individual worm is an independent detector responding to a chemical gradient and that treating each animal as an independent actor in a simple endpoint assay is a useful representation of the biology of chemotaxis. In our “platform and performance” section, we expanded the discussion to emphasize this point and provide evidence that average worm position (derived from treating each worm as an independent measurement) and chemotaxis index (derived from treating each population as an independent measurement) are similar enough to be interchangeable in practice. This analysis is presented in Figure 3, which shows that the two measures are tightly correlated (R = 0.996). Additionally, the practice of computing a single metric (chemotaxis index) is rooted primarily in convenience and there is scant evidence that animals respond as a group.*

*Systematic variability in assay conditions*

*There are many potential sources of variation associated with all behavioral assays. In building this platform, we endeavored to minimize sources of non-random (systematic) variance. For instance, compounds are dispensed using electronic multichannel pipettes, SM identity is masked from experimenters, and compound position is randomized. All assays are conducted at constant relative humidity. Biological replicates are tested on different experimental days. Despite these and other efforts, it is not generally possible to eliminate all sources of systematic variability. As suggested by the reviewer, it is useful to evaluate the impact of such effects, however, by examining the average response on an assay-by-assay basis. Accordingly, we have added these measurements to the plots in Figure 2.*

*REVISION: We have revised the text in the Methods (lines 387-389) and Results (lines 577-581) sections to clarify our approach and now show the average position measured for each of the 16 replicates shown in Figure 2. Additionally, we computed the difference of the mean values (± 95% CI) treating each assay replicate as the fundamental unit of measure. The mean effect size determined by pooling data across the 16 replicates is indistinguishable from that found without pooling (see Table below), except that the 95% CI are narrower in the former case. Such a result is consistent with the idea that pooling increases statistical power with minimal, if any distortion. This analysis is added to Figure 2D, 2E and the legend for Figure 2.*

|  |  | **Analyzed by replicate**  ***N=16*** | | ***Pooled across replicates***  ***n > 2457 worms*** | |
| --- | --- | --- | --- | --- | --- |
| **Control** | **Test** | **Mean difference** | **95% CI** | **Mean difference** | **95% CI** |
| DMSO:DMSO | DMSO:Water | -0.84 | [-2.70, 1.23] | -1.20 | [-2.00, -0.40] |
|  | DMSO:Empty | -0.06 | [-2.99774, 4.03] | -1.03 | [-1.79, -0.27] |
|  | Empty:Empty | 1.62 | [-0.22491, 3.70] | 1.43 | [0.66, 2.21] |
|  | Isoamyl alcohol | 7.50 | [4.16, 11.00] | 7.55 | [6.65, 8.45] |
|  | Diacetyl | 9.65 | [6.38, 13.05] | 8.70 | [7.89, 9.54] |
|  | 2-nonanone | -5.45 | [-8.03, -2.90] | -5.07 | [-5.88, -4.28] |
|  | 1-octanol | -6.80 | [-9.24, -4.10] | -6.66 | [-7.40, -5.88] |

##### Large screens require multiple hypothesis testing. There are now many good techniques for accounting for large numbers of hypotheses in a thoughtful way. For example, by controlling the false discovery rate:

Benjamini Y, Hochberg Y. 1995. Controlling the False Discovery Rate: A Practical and Powerful Approach to Multiple Testing. Journal of the Royal Statistical Society Series B (Methodological) 57:289-300.

Storey JD, Tibshirani R. 2003. Statistical significance for genomewide studies. Proceedings of the National Academy of Sciences 100:9440-9445. doi:10.1073/pnas.1530509100

Since this work is presenting a new type of screen, it is especially important that the authors explicitly discuss multiple hypothesis and set an example for how to interpret them in a statistically rigorous way.

*RESPONSE: We are grateful for this comment as it has helped us to grapple more deeply with the statistical approach. We have studied the papers noted by the reviewer and consulted with co-authors and colleagues with expertise in computation in composing this revision. Our goal is to improve how we justify and explain this approach to readers. It is our fervent hope that others will adopt not only the hardware, wetware, and software we present in this manuscript, but also this analytical approach.*

*Deciding what is worthy of future study. Instead of basing our assessments on binary null-hypothesis significance testing (NHST), we relied on estimates of effect size obtained via bootstrapping methods (*aka *monte carlo simulations). This “estimation statistics” approach has been recommended in editorials published in eNeuro (Bernard, et al, 2019 eNeuro; Calin-Jageman, 2019 eNeuro), Nature Methods (Claridge-Chang & Assam, 2016) and in the Journal of Physiology (Williams, Carson, & Tóth, 2023, J Physiol) as an alternative to NHST. In brief, the estimation approach computes the difference of the mean values between test and control groups by resampling the data 1000s of times. This yields a distribution of the likely true values of the difference of the means, which corresponds to the likely size of the effect of the test condition and is termed the “mean difference”. As noted in the manuscript, we used the DABEST package to perform this analysis, generating shared-control plots (Ho et al, 2019, Nat Methods). Unlike the source data (worm position), this distribution is guaranteed to be normal due to the central limit theorem. In the context of SM screening performed in this study (Figure 5), we considered any test condition for which the 95% confidence interval of the mean difference excluded zero to be of potential interest for subsequent study. This is equivalent to accepting p<0.05 as worthy of further study under conventional NHST (Cumming, Geoff, and Robert Calin-Jageman. 2017. Introduction to the New Statistics: Estimation, Open Science, and Beyond.) and a consequence of this design is that we are likely to have conducted additional studies of some compounds that generated false positives in our screen. Prompted by the reviewer, we applied the Benjamini-Hochberg correction to these data with an FDR of 5% and found that after correction, we would have considered 38 instead of 41 SMs as worthy of further study. In other words, this analysis suggests that only 3 of 41 (or 7%) SMs are likely to be false positives.*

*REVISION:*

*To aid readers, we added text to the Methods (lines 408-418) discussing the estimation statistics approach and the relationship between the 95% confidence intervals for mean differences and conventional null hypothesis significance testing. We also introduced this concept in the Results section (lines 655-659) and discussed the outcome of applying the B-H correction to the screening data (lines 659-661). The exact p values and B-H corrections are presented in a revised S2 Table. As noted above, 3 of the 41 chemoactive SMs were identified as putative false positives by this statistical method: oleanolic acid, sabinene, and sinomenine hydrochloride. Testing these compounds against ion channel mutants, however, delivered evidence that two these compounds might well be chemoactive. In particular, responses to sabinene and sinomenine hydrochloride (but not oleanolic acid) differed in wild-type and anosmic tax-4;osm-9 mutants, indicating that these compounds are bona fide, if weak attractants (lines 743-751). When we tested SMs against single tax-4 and osm-9 mutants, we also found that tax-4 mutants displayed weak attraction to oleanolic acid (lines 761-767). Thus, it seems that distinguishing between compounds that have no effect on wild-type C. elegans and those that generate no NET effect may rely not only on proper corrections for multiple statistical testing, but also on secondary genetic screening.*

*We incorporated additional statistical testing to this revision, using a two-factor design to evaluate the effect of mutation on the response to chemoactive compounds. The two factors under consideration are SM identity and genotype and the computation generates a metric for each SM that is the difference of the differences, or delta-delta. This approach is similar to an ANOVA but does not require that the underlying data be normally distributed or that variances among groups are similar. Each SM associated with delta-delta distributions that* ***exclude zero****, is considered to evoke responses that are sensitive to mutation. Conversely, SMs associated with delta-delta distributions that* ***include zero*** *correspond to cases in which mutation does not significantly alter the responses. This computation is described in the Methods section (lines 419-427) and the results are displayed in revised Figure 6A. We use the delta-delta values as a tool to evaluate how responses to each SM are affected in tax-4;osm-9 double mutants, tax-4 single mutants, and osm-9 single mutants. Figure 6B uses these values to place SMs in a three-dimensional space.*

Clarification on scientific conclusions

Chemotaxis without capturing worms: classic chemotaxis assay captures worms around the droplet with sodium azide. Given that the arena is smaller and does not capture worms on either side, the authors should comment on how this might or might not affect the chemotaxis index. More specifically, there is a chance that preference or valence may not be consistent across 1hr. See for example this instance of valence changing on ~1hr timescale, which probably deserves a mention: Nuttley et al., 2001
 <https://www.ncbi.nlm.nih.gov/pmc/articles/PMC311371/>

*RESPONSE: This comment has three parts that we address below*

1. *How might the absence of sodium azide affect results?*

*It is true that much of the published literature includes studies that use sodium azide to capture worms near the test and control droplets. This practice makes it practical to manually count worms following the assay time period. Since our platform does not rely on manual counting, however, sodium azide is not required. Our experimental design (imaging plates with a flatbed scanner + software to locate worms) allows for reproducible chemotaxis assays in the absence of sodium azide. It is worth noting that sodium azide is an inorganic compound that has long been known to be toxic to humans and animals and is a highly reactive chemical (e.g.* [*Chang & Lamm, 2003*](https://pubmed.ncbi.nlm.nih.gov/12851150/)*;* [*Tat et al, 2021*](https://www.ncbi.nlm.nih.gov/pmc/articles/PMC8349855/)*). By omitting sodium azide, we improve safety for human experimenters, reduce the chances of a chemical reaction between test compounds and sodium azide, and avoid including a chemical unlikely to be present in natural environments. For these reasons, we argue it is both practical and preferable to omit sodium azide from our assays. Despite this difference in experimental design, our findings agree with responses reported in the literature from assays conducted in the presence of sodium azide for the attractants, isoamyl alcohol and diacetyl, and the repellants, 2-nonanone, and 1-octanol (see Fig 2).*

2. *What is the impact of the arena size?*

*Classical chemotaxis assays are performed in a variety of chambers, including 6-cm and 10-cm Petri dishes. In our work, each assay arena is 65 mm from test to reference chemical and this dimension is only slightly larger than the diameter of a 6-cm Petri dish. Thus, we do not think that the arena size has a significant impact.*

3. *What is the impact of the 1-hour assay time?*

*In this study, we prioritized the ability to screen a large panel of small molecules over exploration of variables that might affect response valence, including assay duration. Figure 1 in Nuttley et al, 2001 shows that valence varies over time for one condition (100% benzaldehyde), but not for others (1% benzaldehyde, 1% isoamyl alcohol, 100% isoamyl alcohol). Thus, Nuttley et al show evidence that response valence can vary over time, they also show that this response pattern is not a general property of attractants and is concentration-dependent. While our platform could be adapted to investigate the time-dependence of valence across a large panel of chemoactive compounds in the future, such studies are beyond the scope of this work.*

*REVISION: To address these items, we made the following revisions to the text:*

1. *Revised the Methods section (lines 244-245) to make it clear to readers that we did not add sodium azide to test SMs or to solvent (DMSO).*

*Noted in the Results section (lines 510-511) that it was not necessary to trap animals using sodium azide because of fast endpoint imaging.*

2. *Revised the Methods section (lines 200-201) to note that the apex-to-apex distance is comparable to the practice used in much of the prior literature.*
3. *Revised the Discussion (lines 976-982) to inform readers that we cannot exclude the possibility that the valence we observed during a 1-hour assay might differ from that observed during shorter or longer assay periods and cited the findings of Nuttley et al, 2001.*

##### Graph summary of odor preference: The authors should clarify if the clustering in 7B only uses valence (attraction or repulsion) or if it takes into account the magnitude. And they should clarify how this leads to the groupings in 7C.

*RESPONSE: We thank the reviewer for asking clarifying questions relating to the approach we used to group our chemoactive compounds in Figure 7B. This clustering approach made use of the mean worm position differences derived by taking the difference between a genotype’s mean worm position to a given chemoactive hit and dmso.*

*Upon further evaluation, we opted to modify our approach as follows. First, across all SMs, we used bootstrapping to calculate the difference between each mutant and wild-type and between tax-4 and osm-9 single mutants and anosmic tax-4;osm-9 double mutants. This calculation yielded arrays of delta-delta values for each pair of genotypes. Taking their absolute value generates a valence-agnostic estimate of the size of the effect of mutation on chemotaxis. We used these estimates to cluster SMs into three groups: 1) primarily tax-4 dependent; 2) primarily osm-9-dependent; 3) dependent on both tax-4 and osm-9. The third group was subdivided into SMs for which loss of a single ion channel inverts the response valence. This analysis shows that SMs that depend on a single ion channel effector (TAX-4 or OSM-9) are a rarity — only 8 of 37 SMs qualified in this category.*

*REVISION: The results section has been substantially revised to reflect this analytical approach and its results. These revisions are found under the following subheads: “Anosmic tax-4;osm-9 double mutants are indifferent to SMs” (line 703), “Loss of a single chemosensory ion channel subunit inverts chemotaxis valence” (line 757), “For most SMs, chemosensory signaling depends on both tax-4 and osm-9 ion channel genes” (line 790). Additionally, we revised Fig 7 to show the estimate of the mutant effect size and the groupings based on these values and a classification rubric described in the text.*

#### Limitations

The "Limitations and future research" section mostly lists strengths of the method with almost no limitations. But a thoughtful discussion of limitations is warranted, including but not limited to:

- The assay ignores interactions of the small molecule with the air, solvent or substrate. So different diffusion coefficients, solubility in the solvent, and absorption rate into the gum could all result in changes to chemotaxis unrelated to the animal's intrinsic valence or attraction or repulsion to the molecule.
- The method only reports a static snapshot in time. Therefore it doesn't capture temporal information about the animal's navigation strategy and misses phenomena like habituation or sensitization that may be occurring during chemotaxis. See Nuttley paper mentioned above.

*RESPONSE: Thank you for these critical comments and suggestions.*

*REVISION: We have revised the section on limitations to include the questions you have raised, including variation in physico-chemical properties of the small molecules we tested, and the possibility that the valence we observe is specific to the 1-hour time point. In addition, we discuss solubility issues with DMSO in our methods subsection “Chemical gradient set-up”(lines 232-236) and the second paragraph of our discussion (lines 873-878). All compounds with visible precipitates are annotated with (p) in Table S1.*

#### Minor:

- The equation on manuscript page 12 doesn't render properly and so I cannot see the variables.

*This has been fixed (line 380).*

- The sentence about Spinosad on manuscript p. 26 is missing a main clause.

*Thank you for catching this, it has been fixed (lines 924-925)*

### **Reviewer #3**:

Overall, this is a very good comprehensive study including also innovative technical aspects. Two minor comments that can be easily addressed:

"The primary strategy worms use to accumulate near attractants is to suppress turns (pirouettes) and to increase forward run duration when moving up a chemical gradient [6]. The converse strategy underpins the avoidance of repellents [7]." Indeed, klinokinesis plays the central role during food seeking behaviours od C. elegans; whether it is the primary strategy is not clear and could be debated. We suggest to mention and cite cite some of the major work on klimotaxis i.e., weathervaning, as a paralell strategy.

*Thank you for bringing this to our attention. We added a comment on weathervane-like steering to the introduction (lines 71-72): “They also make use of a weathervane-like steering mechanism to fine-tune chemotaxis* [*(Iino and Yoshida 2009)*](https://paperpile.com/c/sfhDxF/q4w7) *and are seen to modulate their speed in chemical gradients* [*(Albrecht and Bargmann 2011)*](https://paperpile.com/c/sfhDxF/jANC)*.”*

"The platform is simple, delivering all test compounds at a single concentration. This design choice limits the inferences that we might draw regarding response strength and might result in a failure to detect some bona fide responses. It might also affect response valence, since some chemical cues are attractive to wild-type C. elegans at low concentrations and repulsive at higher ones [30-32]. Thus, it is possible that our screen design omits some chemical cues or inverts responses to others."

We suggest to discuss the following point: one more layer of complexity could be introduced through different habituation responses so you might miss compounds like benzaldehyde, where worms seek the gradient peak initially and then crawl away.

Nuttley WM, Harbinder S, van der Kooy D. Regulation of distinct attractive and aversive mechanisms mediating benzaldehyde chemotaxis in Caenorhabditis elegans. Learn Mem. 2001 May-Jun;8(3):170-81. doi: 10.1101/lm.36501. PMID: 11390637; PMCID: PMC311371.

*This exposure-time dependent response was also noted by Reviewer 2. In response, we added a comment to the section in the Discussion in which we address limitations of the approach (lines 976-982).*
