# Supplementary material for: A high-throughput behavioral screening platform for measuring chemotaxis by C. elegans
Source: PLoS Biol. 2024 Jun 27;22(6):e3002672. doi: 10.1371/journal.pbio.3002672 (PMC11210793; doi:10.1371/journal.pbio.3002672)
Supplement: Attachment — Submitted filename: Fryer Response2Reviewers R3.docx [file pbio.3002672.s012.docx]

# Response to Reviewers

We thank two reviewers for their critical feedback, which helped us to improve our attention to potential technical limitations and complications and to statistical analysis. This feedback helped us to improve not only this manuscript, but our analytical procedures for analyzing and interpreting high-throughput genotyping.
